# Data management for distributed computational workflows: An iRODS-based setup and its performance

**DOI:** 10.1371/journal.pone.0340757

**Published:** 2026-01-12

**Authors:** Mohamad Hayek, Martin Golasowski, Stephan Hachinger, Rubén J. García-Hernández, Johannes Munke, Gabriel Lindner, Kateřina Slaninová, Philipp Tunka, Vít Vondrák, Dieter Kranzlmüller, Jan Martinovič

**Affiliations:** 1 Leibniz Supercomputing Centre (LRZ), Bavarian Academy of Sciences and Humanities, Garching near Munich, Germany; 2 IT4Innovations National Supercomputing Center (IT4I), VŠB - Technical University of Ostrava, Ostrava, Czech Republic; 3 MNM-Team, Ludwig-Maximilians-Universität (LMU) München, Munich, Germany; King Fahd University of Petroleum & Minerals, SAUDI ARABIA

## Abstract

Modern data-management frameworks promise a flexible and efficient management of data and metadata across storage backends. However, such claims need to be put to a meaningful test in daily practice. We conjecture that such frameworks should be fit to construct a data backend for workflows which use geographically distributed high-performance and cloud computing systems. Cross-site data transfers within such a backend should largely saturate network bandwidth, in particular when parameters such as buffer sizes are optimized. To explore this further, we evaluate the “integrated Rule-Oriented Data System” iRODS with EUDAT’s B2SAFE module as data backend for the “Distributed Data Infrastructure” within the LEXIS Platform for complex computing workflow orchestration and distributed data management. The focus of our study is on testing our conjectures—i.e., on construction and assessment of the data infrastructure and on measurements of data-transfer performance over the wide-area network between two selected supercomputing sites connected to LEXIS. We analyze limitations and identify optimization opportunities. Efficient utilization of the available network bandwidth is possible and depends on suitable client configuration and file size. Our work shows that systems such as iRODS nowadays fit the requirements for integration in federated computing infrastructures involving web-based authentication flows with OpenID Connect and rich on-line services. We are continuing to exploit these properties in the EXA4MIND project, where we aim at optimizing data-heavy workflows, integrating various systems for managing structured and unstructured data.

## Introduction

Distributed computing in science and engineering has been a great technical and organizational challenge since the appearance of large-scale grid-computing [1] infrastructures such as TeraGrid [2], WLCG [3], and EGI [4]. Many solutions have failed the test of large-scale adoption. However, the idea of using best-suited computing resources across geographical sites for workflows keeps playing a very important role [5]. In line with this, the development of frameworks and infrastructures for distributed data management continues. The EUDAT Collaborative Data Infrastructure has become a major player in data management for European research with its services such as B2SAFE and B2HANDLE [6–8]. The European Open Science Cloud EOSC [9] will then integrate the most promising data-management approaches. Furthermore, European and International Data Spaces [10] are expected to help shape the distributed-data landscape in the coming years.

In this setting, the IT4Innovations National Supercomputing Center (IT4I, Czech Republic) and partners across Europe have developed the LEXIS Platform for distributed computing [11–13]. LEXIS orchestrates workflows across systems at supercomputing centers, including the Leibniz Supercomputing Centre (LRZ, Germany). It leverages heterogeneous computing backends, such as Infrastructure-as-a-Service clouds and classical High-Performance-Computing (HPC) clusters, depending on the use case. The original development project involved three demanding pilot applications, focused on data-intensive simulations and big data analytics in the sectors of weather & climate [14], aeronautics [15] and earthquakes & tsunamis [16].

Computational workflows across different locations need an efficient mechanism for data management, facilitating uniform data access and automated data transfer. For this purpose, a “Distributed Data Infrastructure” (DDI) as a component of the LEXIS Platform was to be set up [17], suitable for generic data from multiple research disciplines. We conjectured *(I)* that an open-source data-transfer and/or distributed data management framework could deliver the needed functionality with limited need for adaptation; *(II)* that our non-functional requirements could thus be satisfied, and in particular that large parts of the available network bandwidth could be utilized for typical data transfers over the wide-area network; and *(III)* that, to this end, performance limitations could be sufficiently understood to mitigate or overcome them with straightforward approaches, for example optimization of buffer size settings or reasonable sanitation of usage patterns.

The construction of the DDI thus involved three challenges: choosing a data management framework, delivering a setup concept, and putting the conjectures *(I)-(III)* given above to a test – which is the focus of this work. As a data-management framework, the DDI leverages the “integrated Rule-Oriented Data System” iRODS [18] with the B2SAFE [7] module. Based on this, it fulfils our functional and non-functional requirements, including basic prerequisites for compliance with the FAIR (Findable, Accessible, Interoperable, Reusable) principles [19] of modern research data management. We present and analyze performance measurements within our large-scale iRODS federation and discuss our learnings from tests and operation. We thus hope to give some guidance for similar applications and to shed further light on the conditions under which iRODS performs well. As LEXIS technology will help to build and operate the EuroHPC Federation Platform [20], our findings are likely of relevance for future European distributed-data and distributed-computing applications.

Below, we first give an overview of related work. We then discuss and illustrate requirements on a DDI supporting workflows as in LEXIS, and lay out how iRODS with the B2SAFE module helps to fulfill these. We explain how we used this framework to build a reliable, federated distributed-data system for the first version of the LEXIS Platform. The article then extensively discusses our experience with the DDI with respect to the conjectures given above. It addresses operational aspects, performance benchmarks, limitations, and directions beyond the original LEXIS setup, as they reflect in our current projects.

## Related work

Distributed data management solutions can roughly be categorized into *i)* low-level file-system and data-management frameworks for storage clusters, *ii)* middleware for unified access to heterogeneous storage systems, and *iii)* solutions loosely federating data systems at the metadata-catalog or data-transfer level. We give an overview and argue that solutions from category *(ii)* lend themselves as a backend for distributed computational workflows. Our exploration focuses on sustainable open-source solutions which were available at the time the first version of the LEXIS Platform was built. We also discuss other data federations of a scale similar to our DDI and work on benchmarks of iRODS systems as relevant to the later sections of our article.

### File-system and management solutions for storage clusters

Ceph [21], EOS [22], GlusterFS [23], HDFS [24], XtreemFS [25] and similar systems manage data distributed over a number of server and disk systems in a data center. The user sees the system as block or object storage – which can be mounted or, alternatively, addressed via interfaces such as S3-compatible [26] endpoints. The solutions are usually strong in guaranteeing storage redundancy, failure resilience and load balancing. Block-wise data distribution or reduplication across physical systems are often used to enhance performance and data safety. With exception of XtreemFS, the systems mostly target a relatively tight integration of the storage elements rather than a federation over wide-area networks between regions and countries. Also, they do not focus on handling metadata or persistent identifiers to support FAIR [19] research data management.

### Middleware frameworks for unified access to heterogeneous storage

Storage middleware frameworks for integration or federation of heterogeneous storage systems include dCache [27], iRODS [18], Onedata [28], Rucio [29] and XRootD [30]. Data on different backends is thus presented through a unified namespace, and cross-system or cross-site data retrieval is facilitated by efficient internal data-transfer mechanisms. With respect to file-system-level solutions, unavailability of sites or higher network latencies can be handled more gracefully. To this end, a typical federation consists of several (remote) storage sites and data is distributed on object, file or dataset level. The systems often support high-level metadata and policy management features, enabling storage tiering or sophisticated data-access control, for example. All these capabilities make middleware frameworks ideal for data federations across computing centers.

### Federation solutions with mere focus on metadata catalogs or data transfer

On a still higher level, data storage can be indexed or federated via repository software or data catalogs. Systems implementing this strategy include Fairspace [31], COLID [32] or Gen3 [33]. Also, Data Spaces [10] – as standardized by the International Data Spaces Association [34] – rely on a federated architecture with metadata catalogs. Other data-management frameworks, such as DataLad [35] or GLOBUS [36] have strongly focused on facilitating the exchange of data or metadata.

In the systems discussed, data transfer is typically triggered by explicit pull or push requests. In contrast, we had envisaged transparent and efficient cross-system access on data through a tighter integration. Therefore, we focused on middleware frameworks.

### Relevant work on large-scale data federations and benchmarking

Large-scale data federations based on middleware often have a background in high-energy physics [37,38]. Such federations typically make use of dCache, XRootD or Rucio [27,29,30]. These solutions show competitive performance in a variety of demanding contexts [39,40], however with data predominantly from one field of science. Thus, the resulting platforms may have a tendency towards higher performance and lower flexibility than our solution discussed here, but can be considered similar overall.

In other fields of research, the value of data federations beyond the facilitation of data transfer [36,41] has been recognized as well [42]. iRODS together with the data replication features of B2SAFE has been used in the CompBioMed project series to set up a data federation across European supercomputing centers [43]. Our DDI for LEXIS follows the same basic principle, but is not domain-specific, and has a focus on elevated availability according to the “High Availability iRODS System” concept [44]. Onedata has been chosen to set up data handling for the cross-site Indigo DataCloud [45], and is used within the EGI Federated Cloud [46]. Although these infrastructures mostly cover cloud-computing applications, the data-federation capabilities are comparable to what we present. Recently, the concept of Data Spaces and the Dataspace protocol [34] have been increasingly adopted [47] as a higher-level federation approach complementary to our work. The European Open Science Cloud [9] is expected to interoperate with European Data Spaces [10].

A number of authors have aimed at assessing the features [48–50] or the performance [40,51,52] of distributed data management frameworks. Due to the heterogeneity and rapid development of such systems, meaningful and precise comparisons are challenging. In our work, we lay out requirements on a data federation in our setting and assess the match with the five different middleware-based systems discussed above. We motivate and discuss our solution, evaluating its performance. Performance benchmarks on iRODS have previously been published by several authors [51,53,54], covering different aspects of the system. However, these works used earlier iRODS versions than we do, and did not focus on testing data transfer performance in a large-scale international federation.

## Basic requirements on a DDI for computational workflows across computing centers

Platforms such as LEXIS [12,13] aim at simplifying the efficient usage of heterogeneous computing systems for science and enterprise applications. They help to automate typical computational workflows, which are sequences or directed acyclic graphs of computing tasks; a simple example would be the sequence: preprocessing – weather simulation – post-processing – output data analytics – visualization. Through orchestration across computing centers, an optimal computing resource can be used for each task. In the LEXIS case, the user supplies a workflow description, necessary applications and input data via the LEXIS Portal [12]. After workflow launch, an orchestration system addresses the infrastructure involved. Input, intermediate and output data are stored on the DDI to ensure data access from all sites and to keep results accessible in the mid to long term. Furthermore, the orchestrator triggers data staging between the DDI and computing systems where computing systems have no direct or efficient access to the DDI (e.g., because of firewalling).

**Fig 1:**
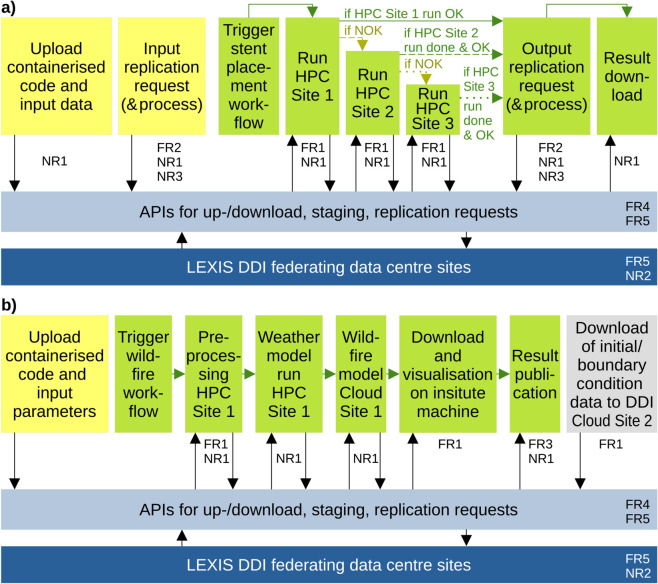
Two example workflows (simplified, read from left to right) that a cross-center DDI as in LEXIS should accommodate. a) stent-placement simulation [56]; b) regular weather and wildfire-risk predictions [13]. Yellow and grey boxes indicate interactive and regularly-executed actions, respectively, while the actual workflow steps are marked green. In case a), runs are attempted on three HPC sites to warrant success in an urgent-computing [55] setting. Functional and non-functional requirements (FR/NR, see text) are related to example functionalities of the workflows. NR2, FR4 and FR5 (see blue boxes) make sure that the DDI offers uniform APIs and authentication across all relevant backends. NR1 and NR3 warrant reliability and performance, most importantly in the urgent-computing case a). FR1 warrants cross-center functionality in both workflows. FR2 facilitates automated cross-center data replication (case a)) and FR3 warrants FAIR Research Data Management (case b)).

This setting is illustrated in Fig 1. The upper part of the figure shows an urgent-computing [55] workflow for simulating stent placement inspired by an earlier demonstration [56]. The example uses input/output data replication in order to attempt to run the simulation on three HPC systems for resilience and to collect the results. The lower part shows a workflow for weather and wildfire-risk simulation, roughly following an example implemented earlier as well [13]. It uses one HPC/IaaS-Cloud site for actual computing, while another site/system is used to regularly download input data.

The setting determines the functional/non-functional requirements (FR/NR) on the DDI as further described below and indicated in the figure. After the requirements collection, we lay out how the requirements motivate our choice of iRODS with B2SAFE module for our first test versions of the DDI (and – owing to the success – for its continued operation). The discussion follows our earlier work [17], but adds performance requirements and an assessment of all middleware systems discussed above.

### Functional requirements

#### FR1: Unified cross-location access to data.

To avoid complications for the orchestration system and for users, the DDI shall implement a unified view on all data in the platform, wherever they may be stored. At the same time, the orchestration system must easily retain awareness of the hosting site for a given dataset to allow for optimized workflow execution.

#### FR2: Implementation of data replication and further storage policies.

A distributed computing platform lends itself [14,16,56] to urgent-computing use cases (see also Fig 1). To avoid unnecessary delays in such a scenario, certain input datasets have to be available on-site at multiple computing centers in the federation.

A DDI accommodating such workloads thus has to automate data replication between the participating computing/data centers, keeping record of the copies stored. This also helps to fulfill expectations on data integrity and availability of academic and commercial users. Furthermore, the DDI should support the implementation of a rich set of storage policies, e.g. for management of tiered storage.

#### FR3: Support for FAIR research data management.

Basic support for the FAIR principles [19] is mandatory in modern research data management, and consequently funding agencies can require implementation of FAIR in research projects using the DDI. Technical aspects of FAIR prominently include the assignment of metadata and persistent identifiers to datasets [19], which the DDI has to support.

#### FR4: Application programming interfaces (APIs) and bindings.

The DDI is to be used via well-defined REST [57] APIs, for example for data up-/download, and staging and replication requests. To implement such APIs [17], the data management system must offer sufficiently rich programming interfaces.

#### FR5: Authentication and authorization infrastructure support.

The DDI system has to interoperate with a common identity and access management (based on OpenID Connect tokens [58] and Keycloak [59] in LEXIS), which helps to implement state-of-the-art security concepts [13], based, e.g., on the zero-trust paradigm.

### Non-functional requirements

#### NR1: Reliability.

The DDI shall be built with redundancy of critical components to offer reliable data management. This helps to create trust in the platform and to avoid a waste of computing time through failed workflows.

#### NR2: Flexibility in backend system usage.

The DDI has to integrate heterogeneous storage backends typically used in production at various computing centers. Integration with both commercial and open solutions should be possible, including, e.g., IBM Spectrum Scale (formerly GPFS [60]) or Ceph [21].

#### NR3: Performance requirements.

Data staging and replication actions in typical applications such as the original LEXIS Project pilots (see also FR2) typically involve datasets with sizes from 10 GB to 10 TB. The time spent for data transfers in workflows should, as a rule of thumb, remain below the time spent for computing. In urgent-computing use cases [14,16], data-transfer times must usually remain in the order of 100 seconds. In use cases involving long simulations on HPC systems [14,15], a data transfer time of one day may be acceptable. In particular, these limits have to be respected for cross-site workflows, while on-site data transfers will typically be faster.

### Choice of system

For the LEXIS DDI as a concrete implementation, we have chosen iRODS with the B2SAFE module. We motivate this choice by assessing the suitability of different middleware frameworks, and by discussing how exactly the solution fulfils our requirements.

#### Suitability of distributed-data middleware.

Table 1 illustrates how well the middleware solutions discussed above match our requirements.

**Table 1:** Requirements on a DDI as in LEXIS vs. characteristics of middleware solutions. “Y” indicates that the solution fulfils the requirement, “N” that it does not. “C” indicates conditional or partial fulfilment.

Requirement	dCache	Onedata	iRODS (+B2SAFE)	Rucio	XRootD
FR1 (unified access)	Y	Y	Y	Y	Y
FR2 (replication)	Y	Y	Y	Y	Y
FR3 (FAIR)	C^1,2^	Y	Y	C^2^	N^3^
FR4 (APIs/bindings)	Y	Y	Y	Y	Y
FR5 (auth. token support)	Y	Y	Y	Y	C^4^
NR1 (reliability)	Y	Y	Y	Y	Y
NR2 (flexible backend use)	Y	Y	Y	C^5^	Y
NR3 (performance)	needs benchmarking^6^				

^1^Handling of user-defined metadata was added in 2020 (DCache 6.2 – extended attributes) when the design decision for using iRODS in LEXIS had already been made.

^2^We have, to the best of our knowledge, no indication that these systems facilitate the acquisition of external persistent identifiers such as DOIs or B2HANDLEs [8,61].

^3^XRootD concentrates on pure file access and handling.

^4^OpenID Connect support was added in or after 2020 [62].

^5^Rucio typically accesses storage through higher-level protocols [63], using XRootD as backend, for example.

^6^Performance must be verified by measurements as in our work; however for all these systems, we had no evidence for critical limitations from the literature.

While iRODS closely matches our requirements, it remains clear that other systems, e.g. OneData, provide similar functionality. Strong points of iRODS are certainly its long and sustained history of development and applications [64], and its architecture federating storage sites without any federation-management server or strong functional interdependencies [18]. Furthermore, in the setting of LEXIS, the vision of embedding the DDI in European data ecosystems has been important. The positioning of B2SAFE with iRODS as a building block of a general-purpose European data infrastructure matches here. We note, however, that other frameworks see a usage within European data-infrastructure projects as well [46,65,66].

#### How iRODS with B2SAFE module fulfills our requirements.

iRODS fulfils FR1 by addressing distributed storage systems through a unified structure of directories and files – dubbed “collections” and “data objects” in iRODS terminology [18]. The top level collections refer to “iRODS Zones” – units in the data federation that can function independently [18], typically corresponding to hosting locations. The framework can react to file-system events (e.g. file creation) by executing freely definable “iRODS Rules” [18], realized via scripts which help to implement policies or functionalities. The B2SAFE [7] module implements iRODS Rules for automatic data replication (cf. FR2) and for the acquisition of persistent identifiers for data through the B2HANDLE [8] service. FR3 is thus met, as iRODS can store user-defined metadata for files and collections. REST APIs and various clients are offered, fulfilling FR4. Via the iRODS-OpenID plugin [67] and some customization work (see section “Access management” below), requirement FR5 has been fulfilled.

Elevated availability (cf. NR1) can be reached with iRODS through well-documented configurations [44]. In addition, we rely on multiple iRODS Zones. The resulting system allows for data mirroring and minimizes the probabilities of data loss or unavailability. iRODS can address a wealth of storage backends, and thus complies with NR2. With our benchmarks, we will test whether sufficient performance is offered to fulfill NR3.

## A DDI concept for LEXIS

We built the LEXIS DDI as an iRODS federation with one iRODS Zone per LEXIS data center. The requirement NR1 motivated us to set up particularly reliable infrastructure within each iRODS Zone. Below, we summarize our concept [17] to facilitate a basic understanding of the system, as relevant for its evaluation.

### High-availability setup for one iRODS Zone

**Fig 2:**
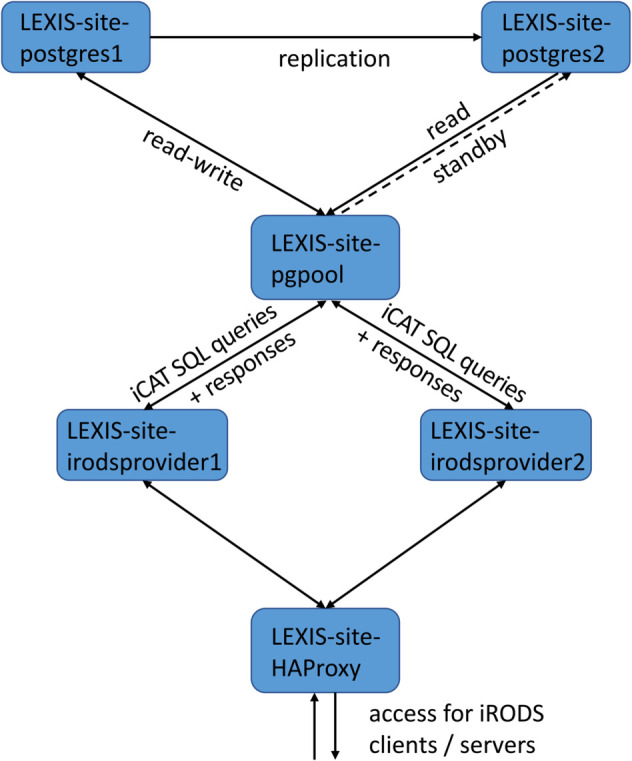
Blueprint iRODS Zone setup. Both iRODS provider servers and database servers as potential points of failure are redundantly provisioned, following [44].

Each of our iRODS Zones has been set up largely following earlier works on the “High Availability iRODS System” concept [44,68,69]. The most critical component of an iRODS Zone is its “provider server” or “iCAT-enabled iRODS server” and the iCAT database connected to it. Via the provider server, virtually all information about an iRODS Zone, including file-system and user metadata, is stored in the iCAT database, for which we use PostgreSQL [70]. We have installed two provider servers and two database servers per iRODS Zone to maximize resilience. The resulting blueprint installation is illustrated in Fig 2.

Our design for the redundant database backend for the iCAT (Fig 2, upper part) uses repmgr and Pgpool-II – see [71] and references therein. repmgr handles live replication between the PostgreSQL instances. Pgpool-II, as ingress on a separate machine, handles request forwarding and manages failovers. Pgpool-II furthermore acts as a load-balancer, using the secondary backend for parallel reading. If the primary backend becomes unavailable, Pgpool-II detects this and performs failover, triggering repmgr to register the secondary database as a new primary. An outage of the new primary in this state would make the database backend unavailable as a whole, as Pgpool-II would have no failover options left. It is thus crucial to bring a failed primary up again in due time, re-synchronizing it using repmgr and re-registering it as a new secondary with Pgpool-II. Within LEXIS, this has been done manually on monitoring alerts. Thus, the original system status has been restored, albeit with database roles switched.

Our redundant iRODS provider servers (Fig 2, lower part) address the Pgpool-II server and are exposed themselves via an HAProxy [72] server as load balancer and ingress. Requests from one IP address to HAProxy have been configured to be redirected to the same default backend to avoid problems with the authentication flow (cf. section “Access management” below).

Pgpool-II and HAProxy are well-proven software products; however they have to be deployed on infrastructure with sufficient availability in order to not constitute a probable point of failure. Managed-server infrastructures within computing centers offer virtual machines with automatic reinstantiation or redundant deployment for such purposes.

### Federation between data centers

The iRODS Zone federation [18] mechanism has been used to implement cross-site data access in LEXIS, while retaining local data-system scopes. Remote iRODS Zone data appears in the collection structure simply under a separate top-level collection, for example /center1Zone or /center2Zone.

Data-staging actions to and from the DDI are handled via a staging REST API, with a backend built on top of iRODS’s interfaces [17], whereas transfer between iRODS Zones can be transparently handled by iRODS on file access through the federation.

### Access management

The LEXIS DDI has been integrated with the LEXIS authentication and authorization infrastructure based on Keycloak [59], an identity and access management solution.

iRODS provides its own authentication mechanism based on username and password, but encourages the use of its plugin architecture [18] for leveraging other mechanisms. The iRODS OpenID authentication plugin [67], in particular, enables the use of OpenID Connect [58] within iRODS. In this approach, the authentication token has been passed in the username field to iRODS, where the field has 1024 bytes in iRODS version 4.2.8. Keycloak, however, uses JSON Web Tokens [73] with a larger size.

We use a combination of pre-authentication and hashing [74] to overcome the size limit. In the architecture of the authentication plugin, the token is sent to a broker service, which validates tokens and stores them in an authentication database. In our solution, the user first pre-authenticates directly with the broker, so that the token is validated and stored. We then modified the Python iRODS Client [75] to hash the token if it is larger than the permitted length. The broker, on the other hand, has been changed to pre-calculate and store hashes of validated tokens and to test received authentication data against both validated tokens and hashes. We have also wrapped the iRODS command line interface (iCommands) to ensure the token remains hashed in the .irodsA file. In the three-way handshake between the client, iRODS and the broker, the iRODS provider server addressed within our redundant system must remain the same. This can be guaranteed in our setup by configuring HAProxy accordingly. In order to have a consistent approach with respect to availability, the broker and its database were set up redundantly as well. We have used setups similar to those described above – in particular a Pgpool-II and repmgr-based setup for the PostgreSQL database of the broker.

LEXIS/Keycloak user identities are mirrored in the iRODS system using the iadmin aua (add user authentication-name) command, and roles are reflected in appropriate group memberships [13,17] using a periodically executed synchronization script.

### Usage of B2SAFE and B2HANDLE

The B2SAFE module [7] is an add-on for iRODS, providing enhanced replication and replica-tracking capabilities. The add-on implements iRODS Rules to put data policies into effect, in particular rules that can be executed for replication of datasets over different backends in an iRODS federation.

**Fig 3:**
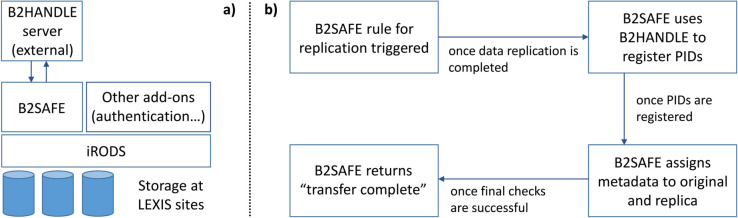
The role of B2SAFE and B2HANDLE for data replication in the LEXIS DDI. The left part (a) of the figure sketches the system, where B2HANDLE is run on a server separate from the DDI (“external”). Part (b) illustrates the replication and replica-registration process.

When run on a collection, the relevant B2SAFE rule will create a replica on the target destination. It will register persistent identifiers for both the source and the replica via B2HANDLE [8], and reflect the relation of the replicas in their iRODS (user-definable) metadata. iRODS can execute rules as a response to events or upon external trigger. In LEXIS, B2SAFE replication is triggered upon request on the LEXIS Portal [12]. For our data-transfer benchmarks, it is run via the command line. The B2SAFE rule execution process and functionality is visualized in Fig 3.

PID assignment via B2HANDLE is used in the LEXIS DDI for datasets wherever needed, in particular in the context of FAIR data handling. B2HANDLE can conveniently be addressed via the EUDATCreatePID function included in B2SAFE, also when not using the replication functionality.

## Performance, limitations and directions for development

Over three years of operation of the DDI enabled us to evaluate the system in depth. Below, we first discuss ease of operation and reliability. We then present benchmark measurements of data-transfer rates and analyze their dependency on dataset size as well as transfer buffer size with the Python iRODS Client [75]. Table 2 shows an overview of these performance tests. Our evaluation refers to the period up to mid 2022, where the original configuration was maintained within the first version of the LEXIS Platform. Besides encouraging results, we also discuss limitations of our concept.

**Table 2:** Overview of performance tests, grouped by figures where the results are shown (second column). Details on the tests and further explanations can be found in the respective sections in the text.

Test	Fig	File Sizes	Number of Streams	Client Buf. Size
LRZ → IT4I: icp^1^	4	5/100/1 000 MB	auto^2^ (1 at 5 MB)	no-client transfer^3^
IT4I → LRZ: icp	4	5/100/1 000 MB	auto^2^ (1 at 5 MB)	no-client transfer^3^
LRZ → IT4I: icp^1^	5	5/100/1 000 MB	auto^2^ (1 at 5 MB)	no-client transfer^3^
LRZ → IT4I: B2SAFE	5	5/100/1 000 MB	auto^2^ (1 at 5 MB)	no-client transfer^3^
LRZ → IT4I: Python^4^	5	5/100/1 000 MB	1	2 MiB
LRZ (Test System) → IT4I (Test System): Python, Buffer 1 MB	6	10/100/1 000 MB	1	1 MB
LRZ (Test System) → IT4I (Test System): Python, Buffer 10 MB	6	10/100/1 000 MB	1	10 MB
LRZ (Test System) → IT4I (Test System): Python, Buffer 100 MB	6	10/100/1 000 MB	1	100 MB

^1^Identical test.

^2^iRODS server and client default to one stream below file sizes of 32 MB and to auto-negotiation above, where the auto-negotiation is likely to yield three streams [76].

^3^icp and B2SAFE “client” applications trigger transfers between resources in the respective iRODS zones; however, in the transfers themselves only the servers are involved.

^4^The Python iRODS Client has been used here to push data (“put” function, see respective section) from LRZ to the IT4I iRODS server.

After 2022, the system underwent some re-design and is currently complemented by database management systems in order to run extremely data-heavy workflows within the EXA4MIND Horizon Europe project (“Extreme Analytics for MINing Data spaces”). We give a glimpse on these developments in the concluding part of this section.

### Experience with the iRODS blueprint setup in operation

In operation, the setup as described delivered the expected functionality, confirming our conjecture *(I)* given in the introduction.

Indeed, the concept prevented unexpected outages of the LEXIS DDI. We are not aware of any user complaints nor of operators noticing major outages while running the DDI as described. However, the relatively complicated concept with six servers for the core functionality of each iRODS Zone has its price.

The redundant PostgreSQL backend showed outstanding stability, but generated substantial system-administration overhead when machines were rebooted for urgent kernel security upgrades. In a typical managed-server infrastructure, for instance at LRZ, such reboots are automated and may happen a few times per year. Once the primary PostgreSQL server reboots, Pgpool-II detects its outage and triggers a failover with de-registration of the instance. As a result, the administrator will find the system with only one PostgreSQL server active, and has to re-synchronize and re-register the other instance, as described in the discussion of our setup. This analogously applies to the authentication-broker system as discussed above.

The duplicate iRODS provider servers in front of the database backend have been set up replicating the internal hostname [69]. To our knowledge, this is necessary as iRODS provider servers are generally aware about their own hostname via the iCAT database, which is shared here. The solution generally worked in our case, but is certainly not best practice from a network and system configuration point of view.

### Benchmarking in real-life configuration

We measured file-transfer performance between the LEXIS sites IT4I and LRZ. Thus, we tested whether iRODS could use the network bandwidth well enough to fulfill our performance requirements (NR3).

We describe the test setup and discuss selected benchmark results below. Further data are included in the Supporting Information. The iRODS version in all tests was 4.2.8; tests involving B2SAFE or the Python iRODS Client were based on the versions 4.3.0 and 0.8.6 of these products, respectively.

#### Test setup.

Files of 5 MB, 100 MB, and 1 000 MB were created on production machines within the LEXIS DDI at IT4I and LRZ. The files were stored on sufficiently fast local storage (e.g. solid-state or RAM drive) for evaluating actual iRODS transfer performance without any influence from file system speeds. What is therefore actually measured is the speed of the wide-area network minus *(i)* performance losses caused by firewall/gateway infrastructure or concurrent network usage, *(ii)* overheads related to the client program and to calls to the iRODS system, *(iii)* overheads due to metadata generation in iRODS data object creation, and *(iv)* overheads of the iRODS protocol itself.

First measurements were taken using icp to copy files. Afterwards, we compared performance measurements using icp, B2SAFE and the Python iRODS Client [75]. Each test was repeated 20 times, and average results are reported and discussed below (the raw data, also for individual repetitions, is delivered with the Supporting Information). Within the series of 20 repetitions, no consistent trends of speedup or slowdown were noted. This agrees with our assumption that read/write processes on our file systems only consume minor amounts of time, which also excludes influences from caching.

#### Results and discussion.

**Fig 4:**
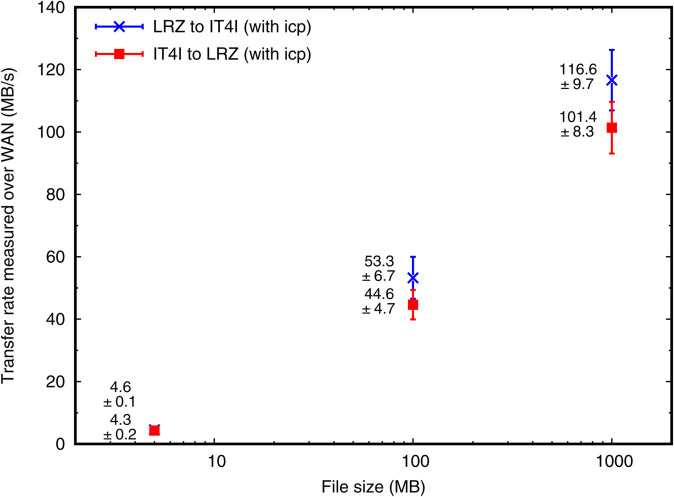
Data transfer rates in the LEXIS iRODS federation (between LRZ and IT4I) with icp. The figure shows average transfer rates and sample standard deviations (from 20 transfer tests per data point shown) in MB/s, using icp on files of various sizes.

Fig 4 shows data-transfer rates for files of 5, 100 and 1 000 MB of size from LRZ to IT4I and vice versa. The copy process has been initiated at LRZ side by issuing the command icp and addressing source and target files in the different iRODS Zones. We expect that issuing the command on the IT4I side would not give a qualitatively different picture. We measure a slight direction-related asymmetry in the transfer rates of 10% - 20%. It is statistically within the sample standard deviation for each file size, but consistent across file sizes. This may be due to various reasons, including the implementation of the actual copy process in iRODS.

For files of ~1 GB of size, decent transfer rates of >100 MB/s are obtained. Within the LEXIS context, these allow for the transfer of typical intermediate-result datasets (10 GB - 1 TB) from one system to the next within a workflow. In particular, smaller datasets can be transferred within ~100 s for urgent-computing cases, and requirement NR3 is fulfilled. Huge input datasets for computations, such as gridded input data for simulation ensembles, are often re-used within several workflows. Such datasets can thus be transferred over a longer, but acceptable period of time, with an effective rate of roughly 10 TB a day.

An important methodical take-home message from the measurements in Fig 4 is the significant speed gain with file size. To explain this speed gain, we take into account typical durations of the file transfers executed: 1 s for 5 MB; 2 s for 100 MB; 10 s for 1 GB. The pure transfer time for 5 MB should be a minor fraction of a second only. This suggests that in the LEXIS DDI ~1 s of time is spent on connecting, writing metadata into the catalog of the target iRODS Zone, and initiating the transfer. Above file sizes of 32 MB, icp is expected to show performance improvements due to the usage of parallel streams [77], so that the times measured for 100 MB and 1 GB reflect maximum reachable throughputs plus initial latencies. Assuming ~100 MB/s to be the throughput (found for the 1 GB test), a duration of 2 s for 100 MB indicates that latencies as found for the 5 MB tests (~1 s) apply to all transfers. This also implies that the transfer rates measured in the 1 GB tests have saturated, i.e. reflect maximum reachable rates, to within ~10%.

Our findings are consistent with those of Hünich & Müller-Pfefferkorn [53], who stated that the iput time for a negligible-size file into an iRODS system can indeed vary between hundredths of a second and a few seconds. Configuration peculiarities in our setup, as our redundancy provisions, firewalls and the cross-country federation, may introduce additional latency with respect to their setup. Bandwidth usage efficiency in iRODS transfers has strongly improved since the measurements of Hünich & Müller-Pfefferkorn in 2010 [53] and Depardon, Le Mahec & Ségui in 2013 [51]. In particular, Russell, Coposky & Keller [54] found that almost full network speed is reached for negligible network latencies, and 50%-80% are retained for network latencies of 50 ms as we typically measure between LRZ and IT4I LEXIS infrastructure. Bandwidth tests between the LEXIS sub-networks at the centers, using iperf3, yielded results of 1.1-1.4 Gbit/s (i.e. 0.14-0.18 GB/s) at the time of our iRODS experiments [78]. This is consistent with the bandwidth through the virtualized project firewall at LRZ as a probable limitation. The >100 MB/s we find for 1 GB files correspond to a ~70% utilization of the network bandwidth, consistent with what one would predict from [54]. The results confirm our conjecture *(II)* given in the introduction, with data rates almost as high as the GridFTP and UFTP mass-data transfer protocols yield them [79,80] over wide- and local-area networks (70%-90%).

**Fig 5:**
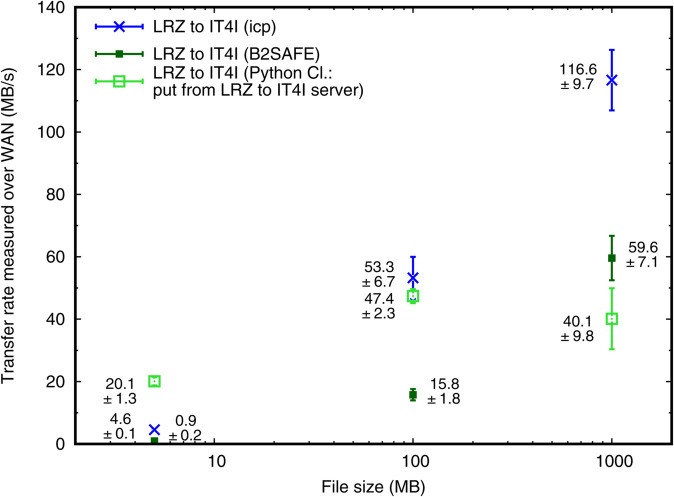
Data transfer rates in the LEXIS iRODS federation (from LRZ to IT4I) with various methods. The figure shows average transfer rates and sample standard deviations (from 20 transfer tests per data point shown) in MB/s, using icp, B2SAFE or the Python iRODS Client on files of various sizes.

Fig 5 compares the speed of transfers initiated with B2SAFE and with the put function of the Python iRODS Client to icp speed in the direction from LRZ to IT4I.

The B2SAFE replication mechanism is based on calling the iRODS-internal msiDataObjRsync and msiCollRsync functions. This presumably yields a similar performance as the irsync command. Relative to the icp transfers, the B2SAFE transfers/replications show a speed of 21%, 30% and 51% with rising file size (5, 100, 1 000 MB). This points towards an additional latency per transfer, probably due to the metadata and persistent-identifier handling of B2SAFE, triggered on each replication. Checks by irsync on target data as part of the synchronization functionality may also contribute to the delay. We have performed additional tests with 10 GB files from LRZ to IT4I (see Supporting Information) and measured an average rate of 130 ± 7  MB/s. This confirms both our assumptions of an additional per-transfer latency in B2SAFE compared to icp (which becomes irrelevant when transferring a 10 GB file) and of speed saturation with icp at a file size of 1 GB (as mentioned above).

The Python iRODS Client shows a completely different behavior. It implements its own put function, which introduces a write buffer that can be manually adjusted. We used a buffer size of 2 MiB in our tests here as a first guess (similar to upstream development). The client, run on a LRZ server machine, then writes out data to the remote (IT4I) iRODS server and target collection in write-buffer chunks as specified. In the version tested, only one parallel transfer stream to the server is used, which certainly limits the speed somewhat with respect to the icp and B2SAFE methods. The results (see Fig 5) indicate a lower overhead at 5 MB file size, but inferior performance for large files. In particular, going from 100 MB to 1 000 MB in file size, performance saturates (or even decreases) instead of an expected increase. Obviously, the network is not fully utilized. This behavior is further investigated below.

### Dependency of performance on buffer size in the Python iRODS Client

Data transfer within an iRODS federation is subject to buffering in all client and server components involved, suggesting potential for speed improvements by optimization or matching of buffer size. Parts of this topic have been discussed by the iRODS team in an extensive whitepaper [54], possibly as a response to earlier claims of a relatively low iRODS performance over networks [51]. Native file transfers between iRODS Zones have – according to Russell, Coposky & Keller [54] – long been optimized in terms of buffer sizes and of negotiation of the number of transfer streams.

In this context, it is illustrative to measure the influence of the internal buffer/chunk size on the performance of the put function of the Python iRODS Client. This aspect has been of practical importance for us, as LEXIS users and the LEXIS Platform are often employing the Python iRODS Client for data access. Below, we again first describe our tests – which have been executed in the scope of a Bachelor’s thesis [52] – and then discuss the results, following the findings in that thesis.

#### Test setup.

A test iRODS system without redundancy was set up between IT4I and LRZ, comprising a few virtual machines in the OpenStack [81] infrastructures at both sites. Performance was then measured as in the benchmarks above, albeit no single-file tests with 5 MB files were made (10 MB were used instead). Tests were continued up to file sizes of 10 GB. A modified version of the put routine of the Python iRODS Client version 0.8.6 was used, where the write buffer size was adjusted to be 1 MB, 10 MB or 100 MB. Transfer speeds measured on virtual machines within an OpenStack infrastructure clearly depend on the infrastructure load. Yet, from runs with different buffer sizes one after another, and consistency checks at different times of the day, a stable overall picture emerged.

#### Results and discussion.

**Fig 6:**
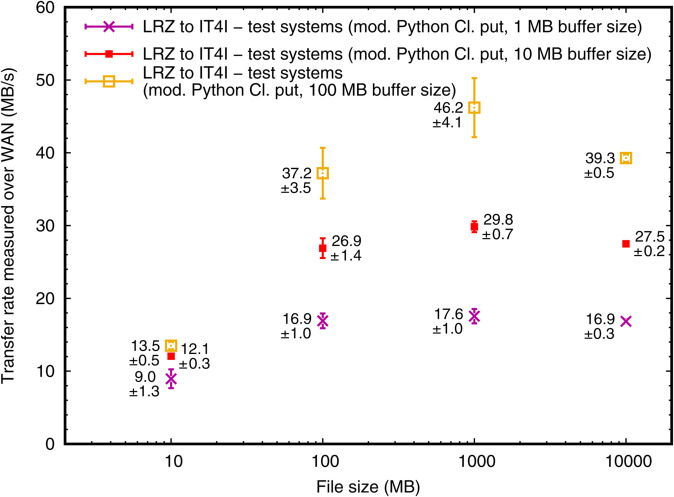
Data transfer rates from LRZ to IT4I in a test iRODS federation, using the Python iRODS Client put routine and various buffer/chunk sizes. The figure shows average transfer rates for various file sizes and sample standard deviations (from 20 transfer tests per data point shown) in MB/s, depending on the buffer size setting in the modified put routine. For this experiment, we have used virtual machines in OpenStack infrastructure on both sides.

The results are shown in Fig 6. The rates here are lower than above, owing to the test infrastructure used. It is evident that only a large enough buffer guarantees optimum transfer speeds. This finding led us to suggest a Python iRODS Client buffer size of at least 100 MB for LEXIS. With this modification, we have roughly doubled transfer speeds for most applications with respect to the original MB-range buffer sizes, confirming conjecture *(III)* from the beginning of this work.

We observe (Fig 6, rightmost data points) that transfer rates with the largest-size files (10 GB) are equal or lower in comparison to those with 1 GB files even when providing a buffer of 100 MB. We may investigate this problematic behavior of the Python iRODS Client, which is somewhat analogous to the observations in the production DDI, further in the future. Possibly, the buffering strategy in the Python code interferes with iRODS-internal or infrastructure-related buffers.

### Limitations of our approach

While our results have been encouraging, a DDI constructed to follow our requirements certainly has limitations inherent to the concept. These limitations are mostly related to the usage of data-management middleware.

Flexibility in federation and data-policy implementation and the extended metadata handling capabilities usually imply performance penalties. These are particularly evident when many small files are transferred (cf. previous sections), as a large part of the middleware-based overheads expresses as waiting times per file-handling action. Furthermore, direct usage of a middleware-based DDI from HPC clusters faces limits by firewalling; for example, no outgoing connection to iRODS ports is allowed on the largest HPC clusters of LRZ. Performance is normally lower than with direct file-system access. This necessitates staging actions to HPC file systems before code runs, where methods have to be optimized and automated within computational workflows.

### Directions for development of the LEXIS DDI

With our experience and benchmarks at hand, and requirements from new use cases, we devise directions for development. The scopes of this are *(i)* the continued maintenance and improvement of the LEXIS Platform beyond the initial project, and *(ii)* projects to extend or complement the DDI functionality.

#### Platform improvements: Ease of DDI maintenance and transfer rate optimization.

As already stated above, the redundant PostgreSQL system generates maintenance work after failovers on a relatively regular basis, and the redundant provider-server setup is not optimum from an engineering perspective. We have thus moved the provider on a single virtual server with sufficient availability, and aim at a redundant PostgreSQL setup with less need for manual intervention. An automated recovery may be achieved via enhanced configuration or using alternative products, such as Patroni on Kubernetes [82,83].

Furthermore, the iRODS consortium has released a new interface for iRODS exposing a well defined HTTP-based API [84] with native support of OpenID Connect. As part of our efforts in the EXA4MIND project, we have updated the LEXIS DDI to use this iRODS HTTP API instead of the native iRODS protocol. We will thus be able to avoid usage of the iRODS OpenID authentication plugin and related middleware, further simplifying maintenance.

To enhance data-transfer rates, a simple, but important measure is to avoid usage patterns involving many small files. This had already been addressed in the LEXIS project, where data compression via a REST API trigger was introduced [17]. We will continue to analyze the structure of our APIs and of iRODS/B2SAFE in order to optimize the usage of the iRODS system from the client side. At the same time, we are pinpointing network-infrastructure limitations (e.g. overloaded switches or firewalls). A bottleneck affecting speeds and maximum dataset sizes in transfers between the DDI and computing systems has been related to temporary storage: Shared data-staging areas have often been used within LEXIS workflows to overcome connectivity restrictions. Enabling direct transfers here, together with the other measures, should consistently enable cross-system and cross-site transfers of the order of 100 TB within a day. We have been working on this to optimally support application cases of the EXA4MIND project, which use the LEXIS Platform with huge datasets.

#### Functionality enhancements.

We plan to enhance and complement the existing functionalities of the LEXIS DDI in a series of subsequent projects. In particular, the EXA4MIND project aims at an integrated management of structured and unstructured data within a so-called “Extreme Data Database”. Instances of the Extreme Data Database comprise database management systems for structured data, and iRODS- and S3-based object stores. These can be flexibly deployed depending on the application, as EXA4MIND automates installation of data stores as well as data staging between these systems. Therefore, we also have the opportunity to index files on an iRODS or S3 system using a database. Fast search or data subsetting mechanisms can thus be implemented, supported by extended metadata extracted from the datasets. Such functionality is developed within the EXA4MIND “Advanced Query and Indexing System”.

EXA4MIND will furthermore enhance connectivity from LEXIS to European data ecosystems. In particular, we target European Data Spaces [10]. Connectivity to Data Spaces involves the installation of a connector [85] supporting the Dataspace protocol [34]. This may require extensions or mappings of the LEXIS DDI standard metadata set, currently adhering to the DataCite standard [86]. All this will help us to extend the FAIR data capabilities of the DDI as well as its interoperability with enterprise data ecosystems.

## Conclusions and outlook

In this work, we have investigated the setup of a distributed data management backend for computational workflows across geographically-distributed computing centers. We conjectured that we could fulfill typical requirements for such a setting with an open-source product and limited customization, that network speeds could largely be utilized, and that we could understand the system well enough for basic performance optimization. This was confirmed by our experience setting up and evaluating the Distributed Data Infrastructure for LEXIS [12], a versatile cloud-HPC-big data workflow platform federating supercomputing centers.

Our data backend for LEXIS is based on iRODS and on B2SAFE [6,7,18]. The choice of the system has been motivated by a requirements analysis and a focus on an optimum immersion into the European data landscape. Our setup follows earlier work on the “High Availability iRODS System” concept [44,68,69]. It warrants elevated availability through redundant deployment of the central server components of each iRODS Zone. LEXIS-specific challenges during the setup have included the integration with Keycloak [74] for authentication, and the federation of multiple computing centers over the wide-area network. Holding metadata, our system works towards compliance with the FAIR principles of research data management [19]. B2HANDLE [8] is used to add persistent identifiers to datasets. B2SAFE enables users to request data replication across sites where appropriate.

Performance benchmarks of the system have yielded sustained data-transfer rates beyond ~100 MB/s between our sites in Czech Republic and Germany. Our measurements complement and confirm earlier work [51,53,54], which has used older iRODS versions and has not focused on the setting of a large-scale federation. A typical waiting time of one second beyond the mere transfer time is observed for cross-site data transfers in the DDI. We have uncovered significant differences between data transfer speeds with icp, B2SAFE and the Python iRODS Client. B2SAFE showed more strongly decreased rates at small transfer sizes. This pointed towards additional latencies of about a second, caused by a more elaborate management of metadata, persistent identifiers and synchronization. The rates achieved with the Python iRODS Client remained relatively low at large dataset sizes, a problem which needs further investigation. We have, however, already been able to roughly double these rates, optimizing the buffer-size settings within the client. Our results for large files and optimum conditions comply with earlier work [54], where it was shown that iRODS transfers can utilize a large fraction of the network bandwidth when correctly configured.

The first years of operating the DDI have met our expectations; nevertheless, limitations and directions for further improvement were identified. The transfer of many small files arguably challenges complex cross-site data systems by construction. This has been addressed by implementing automated compression mechanisms. To reduce workload on the operators, we aim at an enhanced redundancy setup with less need for manual intervention. We intend our work to give a general guidance for setup and utilization of geographically distributed data stores, conveying a feeling for possibilities and optimization strategies.

Within the EXA4MIND EU project on Extreme Data analytics, and LEXIS Platform operation, we continuously improve our systems. Staging mechanisms will be offered for the DDI to interoperate with local databases and object stores, adapted to applications with structured and unstructured data. In the DDI itself, we are eliminating data-transfer bottlenecks and enabling direct data streams between our computing and data-management systems. The resulting data ecosystem will remain interoperable with EUDAT. In the mid to long term, we aim at offering enhanced connectivity to European Data Spaces as well as other European and International data platforms.

## Supporting information

S1 Dataset Measured data from our speed tests. This paper is distributed with data from our speed measurements in csv-table format, packed in a zip file (S1_dataset.zip), as supplementary information. The supplementary data go beyond what has been shown in the figures, with details and further explorative measurements. The general formatting paradigm of the csv tables is as follows:the column separator is a single tabulator sign;the first row contains column headings;the first column contains run ids or experiment descriptors (where average values and sample standard deviations, where calculated, are denoted as “Average” and “Stddev”, respectively);strings are not quoted and do not contain spaces; andall cells but those of the first column and row contain values in MB/s; the decimal sign is a point.
The data in the eight files contained in S1_dataset.zip is as follows:(A) Dataset_hayek25_TestA_cf_fig23_icp_LRZ_to_IT4I.csvContains the speed-test results corresponding to the “LRZ to IT4I (with icp)” part of Fig 4 (note: these data/plot values are repeated in Fig 5). The data columns contain measured values for a file size of 5, 100 and 1 000 MB, respectively. Before the file provides the mere aggregated data for the figures (average, sample standard deviation) in the last two rows, it provides the data from all 20 runs (as executed to obtain average and sample standard deviation) in the first 20 data rows.(B) Dataset_hayek25_TestB_cf_fig2_icp_IT4I_to_LRZ.csvIs structurally equal to file A), but contains the data for the opposite transfer direction (IT4I to LRZ), used in Fig 4.(C) Dataset_hayek25_TestC_cf_fig3_B2SAFE_LRZ_to_IT4I.csvContains the speed-test results corresponding to the “LRZ to IT4I (B2SAFE)” part of Fig 5. While the file is structurally similar to file A), note that it contains one more measurement row for a file size of 10 000 MB to verify the maximum speed and speed saturation behavior as described in the main text.(D) Dataset_hayek25_TestD_B2SAFE_IT4I_to_LRZ.csvContains speed-test results corresponding to those in file C), but in the opposite direction (IT4I to LRZ with B2SAFE – not shown in the figure) and only extending to file sizes of 1 000 MB as in the tests A) and B).(E) Dataset_hayek25_TestE_cf_fig3_PyPut_LRZ_to_IT4I.csvContains the speed-test results corresponding to the “LRZ to IT4I (Python Cl. ...)” part of Fig 5. The file is equal to file C) in structure.(F) Dataset_hayek25_TestF_PyGet_IT4I_from_LRZ.csvContains speed-test results corresponding to those in file E), but in the opposite direction (IT4I to LRZ – not shown in figure). Note that these results, for practical reasons, were obtained by executing the get routine on LRZ side (i.e. we pulled data from IT4I side instead of pushing as in all other tests). Thus, the results are methodically not strictly comparable to those of file E).(G) Dataset_hayek25_TestG_cf_fig4_PyModPut_LRZ_to_IT4I.csvContains all speed-test results corresponding to Fig 6, based on our modified version of the put routine of the Python iRODS Client. The data columns contain measured values for a file size of 10, 100, 1 000 and 10 000 MB, respectively. We have included no single-run values here, as the rows are reflecting the buffer-size dependency (average and sample standard deviation for a buffer size of 1, 10 and 100 MB).(H) Dataset_hayek25_TestH_PyModPut_IT4I_to_LRZ.csvContains speed-test results corresponding to those in file G), but in the opposite direction (IT4I to LRZ – not shown in figure). The file is equal to file G) in structure.
(ZIP)
